# The Cognitive Neuroplasticity of Reading Recovery following Chronic Stroke: A Representational Similarity Analysis Approach

**DOI:** 10.1155/2017/2761913

**Published:** 2017-02-08

**Authors:** Simon Fischer-Baum, Ava Jang, David Kajander

**Affiliations:** ^1^Department of Psychology, Rice University, Houston, TX, USA; ^2^Department of Psychology, University of Massachusetts, Amherst, MA, USA

## Abstract

Damage to certain left hemisphere regions leads to reading impairments, at least acutely, though some individuals eventually recover reading. Previous neuroimaging studies have shown a relationship between reading recovery and increases in contralesional and perilesional activation during word reading tasks, relative to controls. Questions remain about how to interpret these changes in activation. Do these changes reflect functional take-over, a reorganization of functions in the damaged brain? Or do they reveal compensatory masquerade or the use of alternative neural pathways to reading that are available in both patients and controls? We address these questions by studying a single individual, CH, who has made a partial recovery of reading familiar words following stroke. We use an fMRI analysis technique, representational similarity analysis (RSA), which allows us to decode cognitive function from distributed patterns of neural activity. Relative to controls, we find that CH shows a shift from visual to orthographic processing in contralesional regions, with a marginally significant result in perilesional regions as well. This pattern supports a contralesional reorganization of orthographic processing following stroke. More generally, these analyses demonstrate how powerful RSA can be for mapping the neural plasticity of language function.

## 1. Introduction

A well-articulated network of cortical regions associated with single word reading has emerged over the past several decades [1–3], with the left ventral occipitotemporal cortex [4] and the left angular gyrus [5] identified as two critical nodes in the reading network. During the acute stage of stroke, damage to these regions is associated with severe impairments in the ability to read (e.g., [6–8]). These severe impairments often resolve during the transition from the acute to chronic stroke, with many individuals shown to at least partially recover over the years following damage (e.g., [9–15]).

These improvements in reading during the natural recovery from stroke have been argued to provide evidence for neural plasticity, with the damaged brain reorganizing to better support the impaired reading functions (e.g., [12, 13, 15, 16]). Neural plasticity could mean a variety of things: from* functional take-over* whereby the function previously performed by a damaged area shifts to a different brain region to* compensatory masquerade,* or a refinement of established but intact cognitive processes to perform a task [17]. Within the context of reading literature, both of these hypotheses have been proposed to account for the acute to chronic improvements following stroke.

According to the* functional take-over* hypothesis there is a region in the patient's brain whose associated function is different than the corresponding region in the undamaged population. The function of that region in the patient more closely matches the function of the damaged region in the undamaged population. As with much of the language recovery literature, there is debate over whether the region that takes over the function is contralesional or perilesional (e.g., [18]). That is, some have argued that recovery of reading is associated with a retuning of the neural response of the homologous right hemisphere regions, such that this region now computes the function normally associated with the damaged tissue in the left hemisphere (e.g., [12, 16, 19]). Others have argued that the retuning occurs in the tissue just surrounding the lesion (e.g., [13, 15]).

Support for the* functional take-over* hypothesis largely comes from fMRI studies of reading in the damaged brain. For example, in unimpaired readers, it is typical for a region of the left ventral occipitotemporal, frequently referred to as the visual word form area (VWFA), to be more activated to words than baseline, with the region's response being case, font, and location invariant [4]. This pattern suggests that the region is involved in processing orthographic information about written words, that is, abstract information about the letter identities in the word and their order. When undergoing task-related fMRI, patients with damage to the VWFA typically show greater activation to words compared to baseline in the right hemisphere homologue of this region (e.g., [13, 15, 16, 19]) and/or in regions just adjacent to the lesion (e.g., [13, 15]). On the surface, the fact that patients show increased activation to words in regions not typically observed in the unimpaired population suggests that a reorganization of cognitive functions has occurred. Specifically, the orthographic function of the damaged region is hypothesized to be reorganized into other regions that do not typically carry out that function (e.g., [15, 16]).

There are alternative explanations for this reading recovery that do not require assuming functional take-over. Others have argued that the residual reading ability following damage to these regions is due to the refinement of alternative neural pathways for word reading that exist even in the undamaged brain, or a type of* compensatory masquerade*. For example, these patients have been argued to rely on the right hemisphere's normal capacity for visual word processing (e.g., [10, 20, 21]) or alternatively on left hemisphere reading pathways that do not involve the damaged regions (e.g., [14]). In these accounts, recovery over time results from participants learning to more efficiently use these alternative pathways, rather than a dynamic change in the neural organization of the reading system. According to the* compensatory masquerade* hypothesis, even after reading has partially recovered, the functions in the undamaged regions of the patient's brain are the same as the functions in the corresponding regions in the unimpaired population.

Changes in the location of word versus baseline activation in patient's brains may also be consistent with the* compensatory masquerade* hypothesis. Increases in activation in the patient could occur even without functional reorganization; instead they may reflect that the patient is engaging neural regions whose cognitive functions have not been altered by brain damage but that are being used in an atypical way for the task of reading (e.g., [22, 23]). For example, the right hemisphere activation may reflect that the word is being processed visually, but not orthographically. The same right hemisphere region may also process visual, but not orthographic, information about written stimuli in the unimpaired brain (e.g., [16, 24]). Impaired readers show greater activation in that region compared to baseline than the control participants because this visual processing is not well suited for word reading. Without the orthographic processes in the lvOT, impaired readers rely more heavily on these visual processes for word recognition, leading to more activation in the region. Alternatively, the right hemisphere activation may reflect engagement of cognitive processes that are not typically involved in reading for unimpaired readers but become part of the reading process after damage, processes like cognitive control, working memory, or response selection.

Support for this* compensatory masquerade* interpretation of changes in activation profile comes from several longitudinal studies of individuals with VWFA damage. Early in the course of recovery, patients show an increase in the activation of the right VWFA activation to words relative to baseline. As recovery continues and reading improves, less right VWFA activation is observed and greater perilesional activation is observed (e.g., [12, 13]). If, over time, the right VWFA takes on the functional properties of the damaged left VWFA, the opposite pattern would be expected, with more activity in the right VWFA as reading recovers.

This problem of interpreting what the meaning of changes in activation can tell us about the reorganization of cognitive functions is a larger problem in language recovery research. Similar fMRI results have been reported across different types of language impairments, with patients showing both greater perilesional and greater contralesional activation than controls (see [18, 25] for discussion). However, as with reading, early stages of language recovery are more linked to contralesional activation, while later stages of recovery are associated with perilesional activation [23]. Further challenging the idea that contralesional activation reflects functional reorganization is the finding that transcranial magnetic stimulation to contralesional areas in aphasic patients has surprisingly been shown to improve language production [26]. This finding has been interpreted to indicate that contralesional activation may reflect the engagement of a dysfunctional process that inhibits the ability to do the task. However, when a second stroke damages contralesional regions, whatever language has recovered is severely impacted, in both language production (e.g., [27–29]) and reading [30], suggesting that the contralesional region had been supporting the residual language capacity following the left hemisphere stroke.

With all of these difficulties in interpreting changes in activation, it is possible that traditional, univariate activation-based approaches to fMRI are not well suited to address issues of the reorganization of function following stroke. An additional concern is that it is not clear that the* functional take-over* necessarily predicts changes in activation between the patient and control populations. For example, both patient and controls may rely on the same brain regions during reading, but the function of the region is different between the two populations. Alternative methods for analyzing fMRI may be better at distinguishing the* functional take-over* and* compensatory masquerade hypotheses*.

The* functional take-over* and* compensatory masquerade hypotheses* clearly make testable predictions, given the appropriate analysis methods. According to the* compensatory masquerade*, contralesional and perilesional regions in the patient's brain should be doing the same cognitive function as the corresponding regions in the control group. According to the* functional take-over* hypothesis, the function of those regions in the patient's brain should be different than the corresponding regions in the control group and more similar to the normal functions of the damaged region. To address this prediction, it is necessary to identify fMRI methods that can decode what function is being processed by a region, rather than just finding differences in activation level. Here, we use a multivariate approach to analyzing fMRI data, specifically representational similarity analysis (RSA, [31]).

Using RSA, we compare word-similarity measures derived from computation models of different reading-related functions to the patterns of activity distributed across a brain region for individual words. Following existing hypotheses about the neural plasticity of reading following damage, we focus on two levels of representation: low-level* visual* representations of the stimulus code all visual inputs into basic visual features such as oriented edges and* orthographic *representations segment words into a sequence of ordered letter identities and abstract away from low-level visual information like case, font, or location. Other levels of representation like* phonological* representations, which encode the sequence of sounds that correspond to the word being verbalized, and* semantic* representations, which denote the meaning of the word, are also involved in word reading but are outside of the scope of the current investigation.

These levels can be distinguished based on which stimuli are represented similarly. For example, the letters d and b are similar to each other at a visual level of representation, while d and D share fewer visual features. In contrast, at an orthographic level, d and D are examples of the same orthographic letter identity, while d and b are not and thus map onto different representations [32].

Several recent studies have used RSA logic to investigate levels of representation in reading in the unimpaired brain. Rothlein and Rapp [32] focused their investigation on the representation of single letters. They found that the left VWFA was selectively tuned to orthographic representations of letters and a right lateralized occipital region is tuned to low-level visual representation of letters. Fischer-Baum and colleagues [24] focused on whole word representations. They found that the VWFA processes orthographic information about words, while the right homologue processes visual information about the same stimuli. In addition, they found that distributed patterns of activity in the left angular gyrus implicates the region in orthographic processing, while the pattern of activity in the right angular gyrus did not correspond to any of the tested reading-related functions. These studies demonstrate how RSA can be used to decode reading-related functions from distributed patterns of activation.

In the current study, we will apply this approach to the question of the neural correlates of reading recovery. Specifically, we report an fMRI study that compares a single case of an individual, CH, with a chronic reading and writing impairment following a hemorrhagic stroke with a control group. Using RSA, we will determine the informational content of perilesional and contralesional regions in both the patient and the controls by comparing the similarity structure of the region's BOLD activation to individual words to a similarity structure predicted by computational models of representations during reading. In this way, we can adjudicate between the* functional take-over hypothesis* and the* compensatory masquerade hypothesis*.

## 2. Case Study

CH was a 52-year-old right-handed male with a left hemisphere lesion resulting from a hemorrhagic stroke in 2008. He started coming to the lab in December 2011, 37 months following his stroke. Data for the current project was collected between 2013 and 2015. Functional neuroimaging data was collected in July 2014. He has a Master's degree in Chemical Engineering and owned his own company prior to his stroke. As we show below, at the chronic stage, CH was able to read some familiar words despite serious difficulties in processing the identities of individual letters. Similar patterns have been reported previously in the literature [33] and have led researchers to posit an alternative reading pathway available to all readers [34]. Alternatively, during the course of recovery, CH's brain may have changed to allow for a different type of orthographic processing not available to other readers. These alternative accounts of CH's impairment are examples of the* compensatory masquerade* and the* functional take-over hypotheses,* respectively.

### 2.1. Lesion Localization

**Figure 1:**
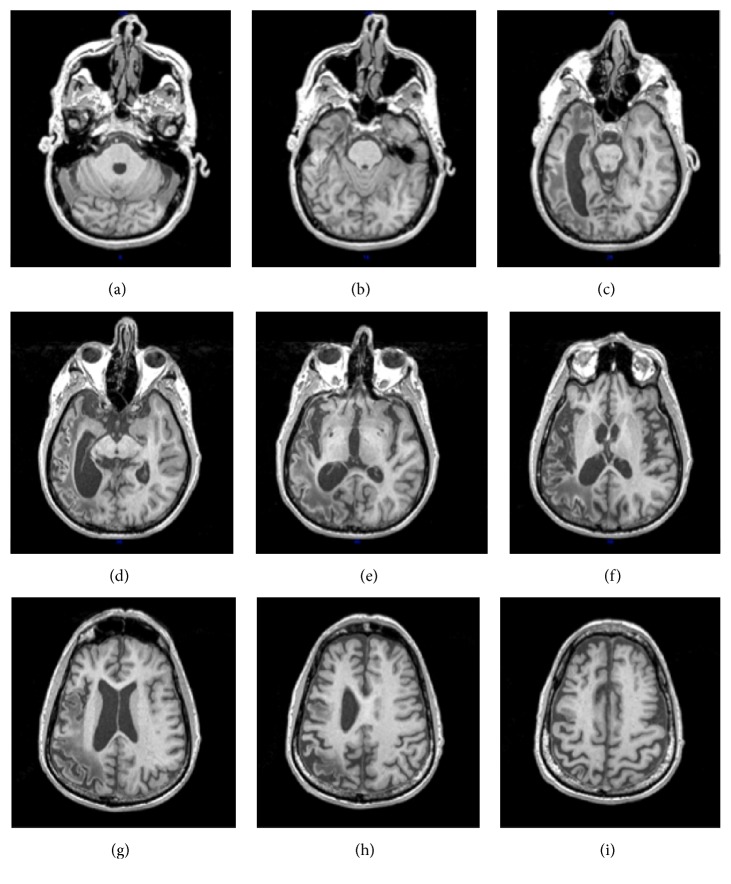
Visualization of CH's lesion from nine axial slices of his T1 scan with 10 mm between each slice going from inferior (a) to superior (i) location.

To localize CH's lesion, a T1-weighted structural image (TR/TE = 8.4/3.9 ms; FA = 8 degrees; matrix size = 256 × 256; FOV = 240 mm; slice thickness = 1.0 mm thick sagittal slices) was obtained. His lesion was segmented manually (following Schnur et al. [35]) and the structural scans, including the lesion mask, were warped and registered to an intermediate template using a symmetric diffeomorphic registration algorithm ([36]; see also http://www.picsl.upenn.edu/ANTS/).  Figure 1 shows nine axial slices from CH's T1 scan. From the intermediate template, scans were then mapped to the “N27 Colin” normalized template in MNI space [37] and then resampled to 1 mm axial resolution using AFNI's 3dresample. Lastly, percent damage was calculated in Automated Anatomical Labeling (AAL) space [38] using VoxBo (http://www.voxbo.org).

CH's lesion included a large portion of the left hemisphere (38% of all left hemisphere voxels). Damage was most extensive in the parietal lobe, including nearly all of the left angular gyrus (90% damaged) and a large portion of the supramarginal gyrus (70% damaged), as well as portions of the left superior (49% damaged) and inferior parietal lobules (67% damaged). There were also extensive temporal damage, in the superior (51% damaged), middle (54% damaged), and inferior (20% damaged) gyri, the temporal pole (26% damaged), and occipital damage, in the superior (35% damaged) and middle (68% damaged) occipital gyri and the fusiform gyrus (18% damaged). Finally, the lesion extended anteriorly to the precentral gyrus (19% damaged) and the posterior portion of the left inferior frontal gyrus, specifically the pars opercularis (30% damaged).

### 2.2. Behavioral Testing

CH received a standard battery of language and cognitive control tasks. His speech perception was assessed as part of a case-series study [39]. CH fell within age- and education-matched controls on both word and nonword minimal pair discrimination tasks, auditory lexical decision, and picture-word matching with auditory foils (*p'*s > .20). In single word, picture matching task [40], CH was always able to correctly indicate when the picture and the word matched and also made no errors when the word and the picture were unrelated phonologically or semantically and only one error with phonological foils. However, he made many false alarms with semantically related foils (29/54, 54% correct), suggesting that he has a semantic impairment or an impairment in accessing semantics from the auditory modality.

There is some evidence that the semantic impairment in speech perception is multimodal. Speech production was also impaired. CH was given the Philadelphia Naming Test [41], on which he was only able to name 87/175 pictures. The majority of his errors (80%) are semantic in nature, evenly split between semantic errors and picture descriptions. He also performed below the control range (47/52) on the three pictures' version of the Pyramids and Palm Trees Test [42]. Therefore, CH appears to have an amodal semantic impairment.

CH also has a striking written language impairment, even on tasks that require processing single letters. CH's visual processing of letters appears to be largely intact. Low-level visual processing was evaluated by a task in which he had to directly copy visually presented letters in the same case. He was nearly perfect at this task (155/156; 99%). He was also able to judge whether a written symbol was a real letter or a pseudoletter (88/88; 100%). Following Schubert and McCloskey [43], this pattern suggests that CH has intact processing up to a visually processing level that stores shapes that correspond to familiar letters. However, on tasks that require orthographic processing of single letters, that is, processing letter identity information that abstracts away from case, font, or modality, CH is quite impaired. He has difficulties naming visually presented letters (26/104; 25%) and copying visually presented letters in the opposite case (57/84, 68%). Therefore, we conclude that CH either has difficulties representing abstract letter identities at an orthographic level of representation that abstracts away from information about case or difficulties accessing these abstract letter identity representations at the orthographic level from the preceding level of representation involved in recognizing familiar letters.

CH also had difficulties processing abstract letter identities from other modalities of presentation. For processing letter identities from the tactile modality, foam letter magnets (approximately 1.75 inches tall) were handed to CH while his eyes were closed. CH had difficulty naming these tactilely presented letters (8/52; 15%) and had trouble copying these tactilely presented letters in the opposite case (10/22; 45%), suggesting that his impairment at the level of abstract letter identities was independent of the modality of input. CH also had difficulties processing letter identities from the auditory modality, for example, with difficulties in writing letters to dictation (13/52; 25%). Given that CH has difficulties processing abstract letter identity information from a variety of modalities, we assume that he has a general impairment in representing abstract letter identity information at an orthographic level of representation.

To assess his ability to read and write whole words, CH was given the same set of 80 high-frequency words between 3 and 7 letters long and 20 pronounceable pseudowords in four tasks: reading aloud, written spelling, oral spelling, and recognition of orally spelled words. This final task has been argued to tap into central reading processes while bypassing the visual system [44]. CH was unable to read or write any of the pseudowords (0/20 in all four tasks), suggesting that reading and writing pseudowords rely on processing individual letter identities at the orthographic level of representation impaired in this participant. He also was unable to orally spell any of the words or recognize a single orally spelled word (0/80 on both tasks) and was only able to correctly write 2 of the 80 word targets (2/80; 2.5% correct). CH's difficulty with processing abstract letter identities had a profound impact on his ability to write words and to recognize orally spelled words, suggesting that these tasks all rely on intact processing at an orthographic level of representation.

CH was also impaired at reading words aloud but was markedly better on this task than the other three, correctly naming 48/80 (60%) of the written words. To further assess CH's ability to read words, he was given the Johns Hopkins University Dyslexia Battery [45]. Overall, he correctly read 89/328 of the stimuli (27%). He was more accurate with words (30%) than nonwords (3%, *χ*^2^ = 30.2, *p* < .0001) and more accurate with high-frequency words (40%) than low frequency words (20%; *χ*^2^ = 7.9, *p* < .01). He showed no effects of spelling-to-sound regularity, reading regular-consistent words (33%), regular-inconsistent (33%), and exceptional words (30%) with the same level of accuracy (*χ*^2^ = 0.1, *p* > .95). This pattern suggests that whatever residual reading ability CH has, it is sensitive to lexical knowledge about the frequency with which words have been encountered, but not by knowledge of spelling-to-sound mappings, which would predict some nonword reading capacity and effects of spelling-to-sound regularity.

Note that this is likely an underestimate of his visual word processing ability. CH's difficulties in picture naming described above suggest language production problems mapping from a semantic system to the phonological system. This language production problem should contribute to CH's difficulties in reading words aloud, particularly since he is unable to read a single nonword [46]. An alternative test of his visual word processing ability is lexical decision, tapping into whether he can recognize that letter strings comprise familiar words. CH was given PALPA 25 which includes 60 words split evenly between high and low frequency and high and low imageability and 60 pseudowords. He was correct on 100/120 (83%) trials, below controls who are nearly perfect on this task, but well above chance. Nearly all of his errors with word stimuli (8/9) were made with words that were of both low imageability and low frequency, indicating, again, that his residual reading ability is limited in its scope to words that are high in frequency and/or imageability.

Further tests were carried out to test whether CH's preserved word processing could be attributed to certain frequent words being recognized perceptually as familiar objects. In one task, we had CH read the same set of 80 familiar words that were presented in UPPER case, lower case, and aLtErNaTiNg case, counterbalanced across a series of sessions. In another task, CH had to make lexical decisions to the same set of 120 words and 120 pseudowords under the same three conditions, counterbalanced across a series of sessions. The logic behind this case manipulation was that CH may have previously seen these words in upper or lower case but would have no previous perceptual experience with these words in alternating case (see [33, 47] for similar logic). If CH's ability to recognize these words depends on familiarity with the perceptual properties of the word, then his performance should be better with upper and lower case words than with alternating case words. Alternatively, if his ability to recognize these words depends on more abstract properties about letter identity and order, then his performance should not be influenced by the case manipulation. In both the reading aloud and lexical decision task, there was no difference in performance for the upper and lower case words than for the alternating case words (reading aloud: combined UPPER and lower case: 56/160 (35%), aLtErNaTiNg case: 27/80 (34%), *χ*^2^ = 0, *p* = 1.0; lexical decision: combined UPPER and lower case: 361/480 (75%), aLtErNaTiNg case: 183/240 (76%), *χ*^2^ = .05, *p* > .82).

Despite severe difficulties in orthographic processing for single letters, CH is able to process some written words. This pattern of performance is not predicted by most theories of visual word processing. Theories typically assume that central reading processes—activating long-term memories of the orthographic representations that correspond to familiar words, semantic representations of the meaning of those words, and phonological representations of pronunciations for both familiar and unfamiliar words—are all necessarily mediated by a level that represents abstract letter identities [43, 48–50]. Furthermore, this pattern is inconsistent with the patterns that are frequently observed in acquired alexia. Most patients with severe problems processing letter identities from visual input also have difficulties in reading words (e.g., [21, 43, 51]), and intact letter identification with impaired whole word reading has been argued to be the basis of the letter-by-letter reading strategy frequently observed in individuals with alexia (e.g., [10, 16]). However, CH's pattern of performance is also not completely unprecedented. Howard [33] reported a similar case of an individual whose abstract letter processing ability was essentially at chance but who was able to read some words (30–40%). While it may be rare, acquired reading deficits in which abstract letter identity processing is more extensively impaired relative to word reading appear to be possible.

Brunsdon and colleagues [34] use this pattern to argue for an alternative route to reading that does not depend on the same level of abstract letter identity representation responsible for processing case-free letter identities. 1 Instead, this theory posits a level of representation that identifies individual shapes as letters in a manner that abstracts away from font but not from case, which precedes the case-free abstract letter identity representations. Furthermore, this theory assumes that there can be direct mappings from these font- but not case-free representations onto stored long-term memory representations of the spellings of familiar words. Assuming damage to the level of case-free representations and this alternative route to recognizing familiar words can explain why CH cannot match letters across case but can read UPPER case, lower case, and aLtErNaTiNg case words equally well.

According to Brunsdon and colleagues [34], this route is available for all readers. Therefore, this argument is a version of the* compensatory masquerade hypothesis*, suggesting that analysis of patient performance reveals a reading pathway that exists even in the undamaged brain. Another possibility is that, over the course of recovery, CH has developed this alternative route, which is not part of the unimpaired reading system. This argument is a version of the* functional take-over hypothesis*, with the damaged brain reorganizing to allow for orthographic processing of familiar words in regions not typically associated with an orthographic function.

Whether CH's residual reading ability reflects* functional take-over* of the reading system or reveals a* compensatory masquerade* remains an open question. To address this issue, CH and group of controls underwent fMRI scanning while reading words. Using representational similarity analysis, we will evaluate whether, contralesionally or perilesionally, CH shows evidence of a change in reading function, specifically a shift in the neural locus orthographic processing, as would be predicted by the* functional take-over hypothesis*.

## 3. fMRI Study

### 3.1. fMRI Methods

#### 3.1.1. Subjects

CH and 20 healthy, right-handed, English-speaking, adult volunteers (aged 18–30) with normal or corrected-to-normal vision participated in the study (controls are the same as those previously reported in [24]). All participants gave informed written consent. The study was approved by the Rice University Institutional Review Board. Participants were compensated $25 for their participation. The entire experiment took approximately 1.5 hours.

#### 3.1.2. Data Collection

Subjects were fitted with a 12-channel head coil in a Siemens 3-T scanner at the Core for Advanced MRI (CAMRI) at Baylor College of Medicine. First, a T1 anatomical scan with 192 1 mm axial slices was collected from each participant. Then, participants underwent twelve function runs to measure BOLD activity during the experimental task. BOLD activity was measured using gradient-echo T2^*∗*^-weighted echo-planar imaging (EPI) of the whole brain. 34 slices were acquired axially, for a voxel size of 3.4375 × 3.4375 × 4 mm (TR 2 s, TE 30 ms, and flip angle 90°).

#### 3.1.3. Task

An event-related design was used. Each of a single list of 40 words (5 proper names, 35 critical words for the analysis) was presented in random order exactly once during each of 12 functional runs. Participants were instructed to press a button whenever a proper name was presented. On a given trial, a fixation cross was presented for 500 ms, followed by a word for 500 m with a trial onset asynchrony of 4 s. Words were presented in all capital letters in Arial font (size 36). On approximately 25% of the trials, a blank screen was presented instead of a word, in order to obtain an estimation of baseline activity [31]. Visual stimuli were presented on a projection screen with an LCD projector and viewed through a mirror attached to the head coil. The 35 critical words were chosen to be intuitively and computationally distinguishable between the four levels of representation tested in the previous paper—visual, orthographic, phonological, and semantic. To adjudicate between the* functional take-over* and* compensatory masquerade hypothesis*, we focus on only the visual and orthographic levels of representation.

### 3.2. Theoretical Similarity Matrices

**Figure 2:**
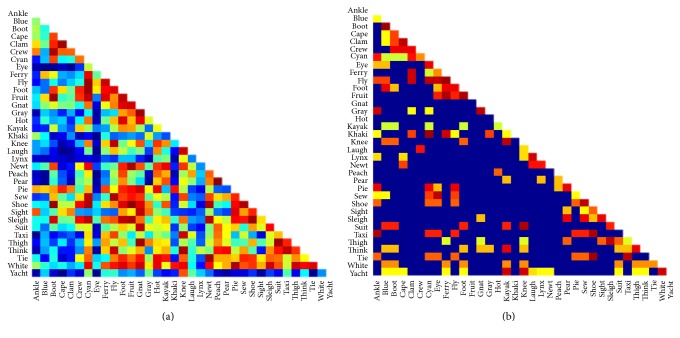
Lower off-diagonal for the two theoretical similarity matrices based on (a) visual and (b) orthographic levels of representation.

For the set of 35 critical words, similarity matrices were calculated based on computationally explicit theories of representation at different levels in the reading system. For the* visual level of representation*, we used a binary silhouette of each word and computed the pixel-wise overlap of the two images [31].* Orthographic similarity* was calculated on the basis of an open-bigram model [52], in which words are represented by multiletter units that reflect ordered pairs of letter identities that are not necessarily adjacent to each other in the word. This type of open-bigram model closely matches the nature of the orthographic code that has been previously proposed to reflect the representational content of the left VWFA [53]. We used the MatchCalculator tool, developed by Colin Davis, to calculate the similarity between words according to this theory (http://www.pc.rhul.ac.uk/staff/c.davis/Utilities/MatchCalc/)^2^.  Figure 2 depicts a visual representation of the off-diagonal of the similarity matrices generated by these different theories of cognitive representation, in which an entry of *i*,  *j* in a given matrix indicates how similar word *i* is to word *j* using a given metric. The Spearman correlation between the two similarity matrices was .12.

### 3.3. Data Analysis

#### 3.3.1. fMRI Data Preprocessing

Data preprocessing was done using SPM12 (University College London, 2012) on T2^*∗*^-weighted functional images. Preprocessing included motion correction, coregistration with the T1, and slice time correction. For the RSA, we chose to forgo spatial smoothing, since differences across adjacent voxels may contain valuable information in the RSA [31]. The segmentation step was carried out, producing forward and backward deformation fields to map to and from MNI space as well as a grey matter mask.

#### 3.3.2. Univariate Analysis

To determine whether the experiment elicited a typical reading network in a standard fMRI analysis, we contrasted all words with fixation, using SPM12. Additional preprocessing steps were applied. Spatial smoothing was done using 8 mm full width at half maximum Gaussian smoothing kernel and the images were warped into MNI space at a resolution of 2 × 2 × 2 mm with 4th-degree B-spline interpolation. For each participant, a 1st-level analysis used a contrast to compare all words, though not proper names, to a fixation baseline. Six motion parameters and scanner drift were included as covariates in the univariate analysis. A 2nd-level analysis used a Crawford's modified *t*-test [54] to determine if the t-maps that resulted from the 1st-level analysis for CH fell outside of the distribution of the control group.

#### 3.3.3. Representational Similarity Analysis

For the RSA, no smoothing or normalization was applied during preprocessing. Beta-weights for each word in each run against fixation were obtained by a general linear model predicting BOLD response, which included the timing of each individual word (modeled as an event) deconvolved with a hemodynamic response function and six motion parameters and scanner drift as nuisance variables. By averaging the beta-weights across 12 trials per subject, we obtained 35 beta-weight maps for each subject, with each map reflecting the brain's response to each word in the experiment. These beta-weight maps were then mean centered within each subject. ROI analyses were applied to these 35 individual-word beta-weight maps for each participant. For each ROI, a vector of beta-weights for the voxels within that ROI was extracted for each of the 35 words. A similarity matrix of word-to-word similarity for this region was calculated based on a Spearman correlation of the beta-weight vectors for each word to every other word. 3 Each entry in the resulting similarity (correlation) matrix therefore represented how similar the distributed pattern of activity within the ROI is between two stimuli. The resulting similarity matrix of pairwise correlations was then compared with visual and orthographic similarity matrices described above, using a Spearman correlation of the off-diagonal values. We refer to the Spearman rho value for the correlation between the brain-based similarity matrix and the visual similarity matrix as the* visual similarity index* and the Spearman rho value for the correlation between the brain-based similarity matrix and the orthographic similarity matrix as the* orthographic similarity index*. For each participant (both controls and CH) and for each ROI, two values were calculated—visual and orthographic similarity indices for the region.

#### 3.3.4. Anatomical ROIs

A challenge to the RSA approach is selecting the appropriate regions of interest for the analysis. One option is data driven, selecting ROIs on the basis of the whole brain analysis, identifying regions in which CH shows more activation than controls and investigating the function of these regions in CH and controls. A second option is hypothesis driven, selecting regions anatomically, based on the hypotheses that reorganization is happening either in tissue just adjacent to the lesion (perilesionally) or in the right hemisphere homologues of damaged regions that are known to be relevant to reading in the undamaged brain. For the current study, we opted for the hypothesis driven approach to selecting ROIs. Because we are investigating only a single case study, the results of the whole brain analysis may be unreliable, with both type 1 and type 2 errors, making it a poor source for selecting ROIs. Additionally, as discussed above, it is not clear that the type of neural plasticity that we are investigating with RSA will result in changes in activation, especially if the function of a region switches from one reading-related function to another. Furthermore, the goal of the current study is to investigate specific claims about perilesional and contralesional reorganization, and there is no guarantee that we will find areas of increased activation in the whole brain analysis for a single subject in either of those critical regions. Therefore, we limited our ROIs to anatomical regions defined on the basis of specific hypotheses about the reorganization of function. Specifically, we looked at three types of region of interest—CH's lesion location in unimpaired participants, contralesional regions of interest, and perilesional regions of interest. We discuss the motivation for each type of ROI below.

One of the goals of the current study is to determine if the function that is normally computed by the region of cortex that is damaged in CH's brain has moved to a different region. Therefore, one ROI involves looking at the information processing capacity of the CH's damaged cortex warped onto the control participants. A lesion mask for CH was traced using MRIcron with “1” assigned to damaged voxels and “0” to other voxels and normalized to the MNI template brain using SPM12 and then warped into each subject's native space.

A second set of ROIs focused on decoding activation in contralesional regions. Specifically, we focus on regions that are (1) damaged in CH and (2) have been argued to have left-lateralized processing of orthographic information in control participants. We will then evaluate whether the right hemisphere homologue of these regions compute different reading functions in CH compared to controls. Fischer-Baum et al. [24] identified orthographic processing in the left VWFA and left angular gyrus (ANG), two regions partially damaged in CH. Therefore, left and right vOT and left and right ANG ROIs were used to analyze whether there is contralesional functional take-over in CH. The left and right VWFA ROIs were 12 mm^3^ radius spheres centered on the MNI coordinates [±45, −57, −12] (based on Talairach coordinates for the VWFA form [55], MNI coordinates based on [56]) and were created using MRIcron [57] and left and right ANG were taken from the Automated Anatomic Labeling (AAL) Atlas [38].

A third set of ROIs focus on functional take-over in cortical regions adjacent to the lesion. Given CH's large lesion, we subdivided his perilesional space into five regions of interests. These masks were created by identifying undamaged voxels from different Automated Anatomical Labeling (AAL) regions. The* fusiform* mask was all of the voxels outside of CH's lesion mask in the left fusiform gyrus. The* inferior temporal *mask was all of the voxels outside of CH's lesion mask in the left middle and inferior temporal gyri. The* medial temporal *mask was all of the voxels outside of CH's lesion mask in the left hippocampus, parahippocampal gyrus, and amygdala. The* anterior temporal *mask was all of the voxels outside of CH's lesion mask in the left superior and middle temporal pole. Finally, the* parietal* mask was all of the voxels outside of CH's lesion mask in the left supramarginal gyrus, angular gyrus, superior parietal lobule, and inferior parietal lobule.

All masks were warped into each subject's native space at the resolution of the T2^*∗*^ image using normalize function from SPM12 and the backward deformation fields produced by the segment function in SPM12 during the preprocessing stage, with nearest neighbor interpolation. Second-level analyses were carried out across subjects for both similarity index values in each ROI. For each ROI, one set of tests focuses on determining the extent to which each ROI computes visual and orthographic information in the controls. A second set of analyses investigates whether the function of the region has shifted to orthographic processing in the CH, relative to controls. Our dependent measure is the difference between the orthographic and visual similarity indices in contralesional and perilesional ROIs. For this analysis, statistical analyses were carried out using a one-tailed Crawford's modified *t*-test [54] under the null hypothesis that CH's difference between the orthographic and visual similarity indices for a given region was not greater than the distribution of the control participants.

## 4. Results

### 4.1. Univariate Analysis

**Figure 3:**
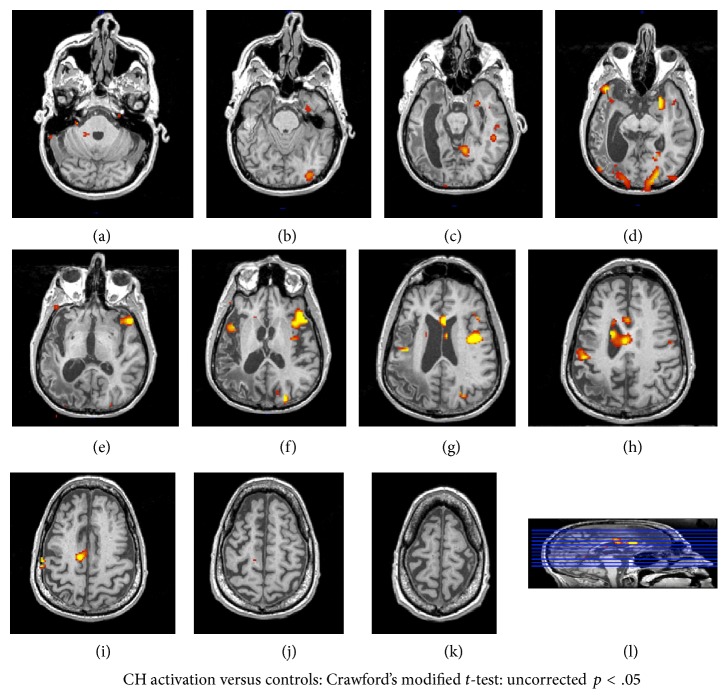
Results of the whole brain univariate analysis identifying regions where CH shows greater activation in words versus fixation than the control distribution plotted in CH's native space (uncorrected* p* < .05).

Figure 3 shows the results of the univariate analysis warped to CH's anatomical scan, with an uncorrected *p* < .05 comparing CH's words versus fixation analysis to the distribution of the controls words versus fixation analyses, using a Crawford's modified *t*-test. Bilaterally, CH shows more activation than controls in a number of regions: occipital cortex (calcarine sulcus and lingual gyrus), the insular and cingulate cortex, and the inferior frontal gyrus. In addition to these bilateral regions of activation, CH showed perilesional activation in the middle occipital gyrus, the posterior middle temporal gyrus and the postcentral gyrus. He also showed contralesional activation to damaged left hemisphere regions typically associated with word reading, including the right midfusiform gyrus and the superior temporal gyrus. Finally, he showed greater activation to words than controls in other right hemisphere regions like the right parahippocampal gyrus and the superior frontal gyrus.

#### 4.1.1. CH Lesion ROI

**Figure 4:**
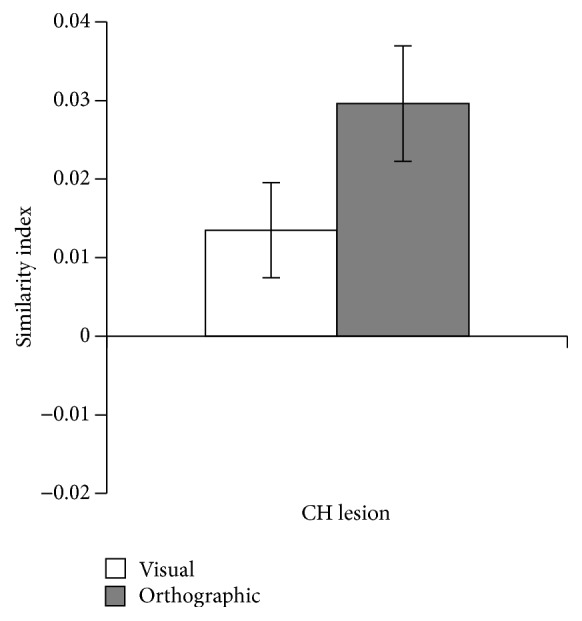
RSA results for the CH Lesion ROI. The graph reports the average (Spearman) correlation between each subject's brain-based similarity matrix with the two theoretical similarity matrices based on computational models of visual and orthographic representation. Error bars represent ±1 SEM.

Figure 4 plots the average of the 20 control participants for each of the visual and orthographic similarity indices for the ROI based on CH's traced lesion. A one-sample *t*-test reveals that, in the CH Lesion ROI, both the visual (.013; *t*(19) = 2.23, *p* < .05) and orthographic (.030; *t*(19) = 4.03, *p* < .001) similarity indices are significantly different than zero. A paired *t*-test indicates that the lesioned region is marginally more involved in orthographic processing than visual processing (*t*(19) = 2.01, *p* = .059).

#### 4.1.2. Contralesional ROIs

**Figure 5:**
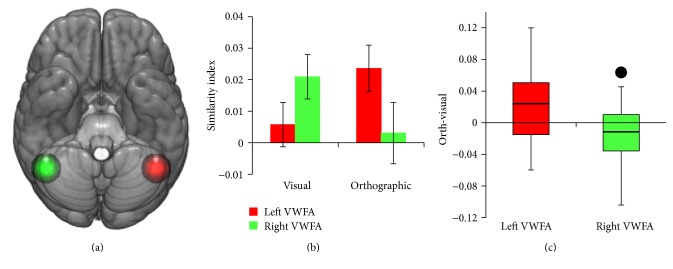
Results of the left and right VWFA ROI analyses. (a) shows the location of the left and the right VWFA regions of interest. (b) plots the average (Spearman) correlation between each control subject's brain-based similarity matrix (separately for the left and right VWFA ROIs) with the visual and orthographic similarity matrices. Error bars represent ±1 SEM. (c) depicts a box-and-whiskers plot for the distribution of the difference between the orthographic and the visual indices for all of the control participants in both ROIs, with the black dot in the right VWFA depicting CH's difference score.

Control results for the ROIs used in this analysis are reported in Fischer-Baum and colleagues [24].  Table 1 reports the range of control orthographic and visual similarity index values for these contralesional regions, as well as the subsequent perilesional ROI analyses, along with CH's value for the orthographic and visual similarity index in each region and CH's rank among the control participants.  Figure 5 shows the results of the analysis for the portion of the left and right ventral occipitotemporal cortex frequently referred to as the visual word form area and its right hemisphere homologue. For controls, a significant interaction between hemisphere and orthographic versus visual representation was observed in this region (*F*(1,19) = 11.3, *p* < .01). Consistent with Dehaene and Cohen [4], in the left VWFA, the orthographic index (.024) was higher than the visual index (.006), while in the right VWFA the visual index (.021) was higher than the orthographic index (.003).

CH's lesion extends into this region in the left hemisphere. Under the contralesional version of the* functional take-over* hypothesis, we might predict that his right VWFA has reorganized, changing its function from visual to orthographic processing [16].  Figure 5(c) presents a box-and-whiskers plot for the distribution of the difference between the orthographic and the visual indices for all of the control participants in both the left and right VWFA ROIs. While, on average, the orthographic index is higher in the left VWFA and the visual index is higher in the right VWFA, it is not true for all participants. The black dot depicts the difference between CH's orthographic and visual indices in his intact right hemisphere. Unlike the majority of control participants, CH shows more orthographic than visual processing in the right hemisphere homologue of the VWFA. CH ranks below all 20 control participants in terms of the visual similarity index and above all but 5 controls in the orthographic similarity index. Using a one-tailed, Crawford's modified* t*-test, we found that CH showed greater orthographic-relative-to-visual processing in the right hemisphere than controls (*t*(19) = 2.09, *p* < .05).

**Figure 6:**
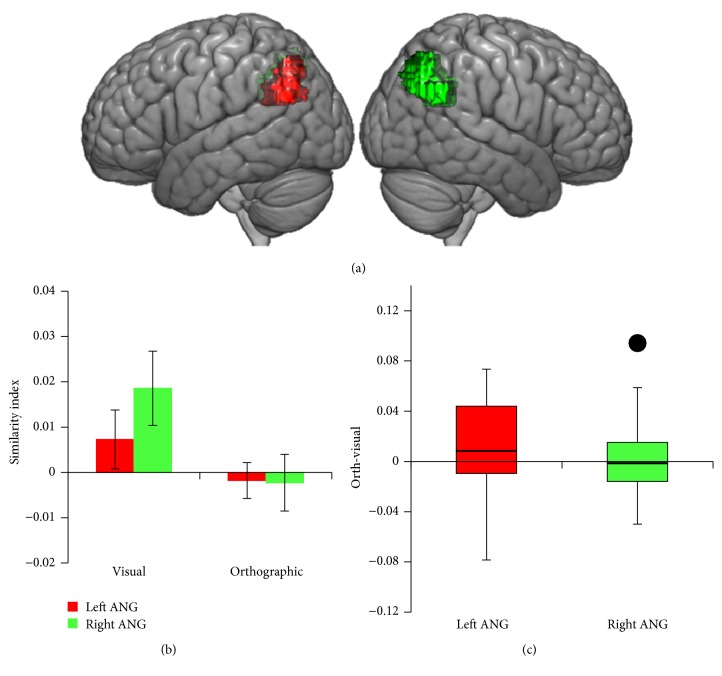
Results of the left and right angular gyrus (ANG) ROI analyses. (a) shows the location of the left and the right ANG regions of interest. (b) plots the average (Spearman) correlation between each control subject's brain-based similarity matrix (separately for the left and right ANG ROIs) with the visual and orthographic similarity matrices. Error bars represent ±1 SEM. (c) depicts a box-and-whiskers plot for the distribution of the difference between the orthographic and the visual indices for all of the control participants in both ROIs, with the black dot in the right ANG depicting CH's difference score.

Figure 6 shows the results of the same analysis for the other contralesional ROIs, the left and right angular gyrus. For controls, neither the left nor the right angular gyrus shows any evidence of visual processing (*p'*s > .27). However, there is evidence for left-lateralized orthographic processing, with the left angular gyrus (.019) having a significantly larger orthographic similarity index than the right angular gyrus (−.002; *t*(19) = 2.45, *p* < .05). As with the left vOT, CH's stroke has damaged a large portion of the left angular gyrus. We analyzed whether CH uses the right angular gyrus to process orthographic information. Figure 6(c) depicts the distribution of the difference between the orthographic and the visual indices for the 20 control participants in both the left and right angular gyrus using a box-and-whiskers plot, with a black dot indicating CH's orthographic minus visual similarity index for his intact right hemisphere. CH ranks below all 20 control participants in terms of the visual similarity index and above all 20 controls in the orthographic similarity index for this ROI. Unlike the control participants, CH had a much larger orthographic similarity index relative to his visual similarity index in the right angular gyrus. This value was significantly above the distribution of corresponding values in the control population (*t*(19) = 3.13, *p* < .01).

#### 4.1.3. Perilesional ROIs

**Figure 7:**
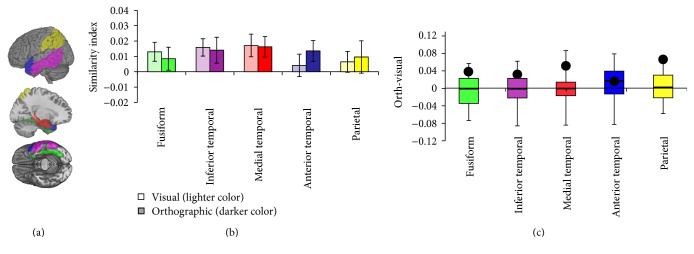
Results of the perilesional ROI analyses. (a) shows the location of the five regions of interest: fusiform (green), inferior temporal (purple), medial temporal (red), anterior temporal (blue), and parietal (yellow) regions. (b) plots the average (Spearman) correlation between each control subject's brain-based similarity matrix with the visual and orthographic similarity matrices. The white box indicates that for all 5 perilesional ROIs the lighter color is the visual similarity index and the grey box indicates that for all 5 perilesional ROIs the darker color is the orthographic similarity index. Error bars represent ±1 SEM. (c) depicts a box-and-whiskers plot for the distribution of the difference between the orthographic and the visual indices for all of the control participants in all five ROIs, with the black dot depicting CH's difference score.

Figure 7 plots the results of ROI analysis for the five perilesional regions of interest, left hemisphere fusiform, inferior temporal, medial temporal, anterior temporal, and parietal regions adjacent to CH's lesion. Using a one-sample *t*-test, the visual similarity index was significantly different than zero for control participants in the fusiform (.013; *t*(19) = 2.10, *p* < .05), inferior temporal (.016; *t*(19) = 2.80, *p* < .05), and medial temporal (.017; *t*(19) = 2.33, *p* < .05) perilesional ROIs, but not for the anterior temporal or parietal ROIs (*p'*s > .35). The orthographic similarity index was significantly different than zero for control participants in the medial temporal lobe (.016; *t*(19) = 2.45, *p* < .05) and marginally different than zero in the anterior temporal lobe (.014; *t*(19) = 1.97, *p* = .064), but not in the other ROIs (*p'*s > .11).

Figure 7(c) depicts the distribution of the difference between the orthographic and the visual indices for the 20 control participants in all five perilesional ROIs, with the black dot indicating CH's difference value. There are marginally significant difference between CH's difference value and the distribution of the control difference values in the perilesional parietal ROI (*t*(19) = 1.73,* p* = .050) and medial temporal ROI (*t*(19) = 1.49, *p* = .076), but not in any of the other perilesional ROIs (*p'*s > .12). The ranking results (Table 1) were similarly ambiguous; CH did not have either the lowest or the highest visual or orthographic similarity index in any of the regions. For the visual similarity index, he fell below the median in the fusiform (17/21), inferior temporal (17/21), and medial temporal (19/21) ROIs, at the median (11/21) for the anterior temporal ROI, and slightly above the median (9/21) for the parietal ROI. For the orthographic similarity index, he fell above the median for all but the anterior temporal ROI, in which his orthographic similarity index was the median value (11/21). His orthographic similarity index in the parietal perilesional ROI was higher than all but two of the control participants.

## 5. Discussion

Following a hemorrhagic stroke, CH was left with a severe written language impairment. Over several years, his familiar word reading improved, though he remained completely agraphic and had residual difficulties in processing abstract letter identity information for single letters. The goal of the current research is to investigate whether his residual reading ability was supported by* functional take-over*, whereby damaged functions have been reorganized into different brain regions, or whether it reflects a* compensatory masquerade*, whereby recovered reading depends on an alternative neural pathway that exists for all readers. By using an fMRI multivariate pattern decoding technique, we demonstrate that CH's brain shows evidence of functional take-over. Most clearly, we observe contralesional reorganization. We looked specifically at two right hemisphere regions that are homologous to the left hemisphere regions that are damaged in CH and have been shown to be important for orthographic information processing in controls. The results of our analyses suggest that CH is now using these right hemisphere regions to process orthographic information in manner that is distinct from the control participants.

As in previous research, we addressed this issue using functional MRI, scanning CH while he read words and comparing his results to a control sample (e.g., [13, 15, 16, 19]). Previous research has used this approach to identify cortical regions that show more of an increase in activation to written words relative to baseline for patients than controls. Taking this same analysis approach with CH, we found a familiar pattern of results. CH showed more activation than controls in a contralesional region close to the right hemisphere homologue of the VWFA, as well as more activation than controls in regions surrounding the lesion in the left hemisphere and additional bilateral frontal activation.

What differences in activation between patients and controls mean in terms of functional reorganization remains an open question [25]. While these activation instances are frequently interpreted as a shift in the locus of a cognitive function, they have also been argued to reflect engagement of processes not typically used by controls [22] or even dysfunctional processing that inhibits the patient's ability to perform the task [26, 58]. We, therefore, used an alternative to these traditional univariate activation-based approaches, a multivariate technique that allowed us to decode function from a distributed pattern of brain activity [31]. Specifically, using this analysis, we compare evidence for orthographic processing and visual processing in different cortical regions.

In unimpaired participants, there was clear evidence that CH's damaged cortex is involved in both visual and orthographic processing when reading words but, tentatively, is more involved in orthographic processing. It is worth noting that this analysis is carried out over a large region of interest and we are not concluding that this entire region is involved with both visual and orthographic processing of written words. Instead, it is likely that different subregions of the ROI are responsible for processing visual and orthographic information about written words. For example, CH's lesion does include damage to the middle and superior occipital gyri as well as the inferior temporal lobe and the angular gyrus. It is possible that visual processing in the occipital subregions of the large lesion ROI are responsible for the conclusion that the lesion ROI is involved in visual processing of written words, while the temporal lobe and angular gyrus subregions of the lesion ROI are responsible for the conclusion that the lesion ROI is involved in orthographic processing of written words.

Reorganization of the damaged orthographic function is clearest in the contralesional ROIs. We looked at two left hemisphere regions that are largely damaged in CH and in which controls show more evidence of orthographic than visual processing (left VWFA and left ANG). In the right hemisphere homologues of those regions, controls show either the opposite tendency (right VWFA) or no difference between orthographic and visual processes (right ANG). Unlike controls, CH shows a greater tendency to process orthographic information than visual information in both of these contralesional regions. CH uses these contralesional regions to compute a different function than that same region in controls. Furthermore, the function that CH is computing in these regions is similar to the function that controls are computing in tissue that CH no longer has following his stroke. This pattern of results is precisely the pattern that would be predicted if there is contralesional functional take-over, whereby the right hemisphere takes over the function of the damaged left hemisphere. At least in right hemisphere regions contralateral to the dyslexia-inducing lesion, there is evidence for functional reorganization in CH, with the region now processing orthographic information at least for familiar written words. Recall that CH's is still impaired at tasks, even with single letters, that require crossing case or modality. However, he remains relatively good at reading familiar words even when they are presented in an unfamiliar manner, like with alternating case. Therefore, we conclude that the orthographic information being processed in his right hemisphere supports the recognition of individual letters abstracting away from certain visual properties of the stimulus like font and location on the screen, but not other visual properties, like case.

In terms of the ranking data, the results were the clearest for the right ANG; CH has the highest orthographic similarity index value in this region relative to the 20 controls and the lowest visual similarity index value. However, one challenge in interpreting this pattern is that it is unclear what type of reading-related computation control participants are doing in this region. Specifically, it is possible that controls have an alternative reading pathway that goes through this region but that does not respond obligatorily during the fMRI task because controls read via a different pathway. If so, the increase in the relationship between the pattern of activity and the orthographic similarity metric in this region for CH might reflect the use of a preexisting system that is silent in the controls. The same argument cannot be made for the right VWFA. In control participants, the pattern of activity in the right VWFA correlates with the visual similarity metric, indicating that this region is engaged during reading and is doing visual processing. CH shows less visual processing and more orthographic processing in this region than controls. For this region, therefore, CH shows a clear shift in the normal reading function which cannot be explained by assuming a preexisting, but silent, reading pathway with orthographic processing in the right VWFA.

There is more limited support for this type of neural plasticity in perilesional regions. Regions that are superior/posterior to the lesion, in the perilesional parietal lobe, and medial to the lesion, in the medial temporal lobe, have a marginally greater tendency to process orthographic information in CH than in the controls. We therefore do not want to make any strong conclusions about whether or not CH shows perilesional reorganization of function. The role of contralesional and perilesional regions in recovery is an open question in the neural plasticity of language [18, 25]. In reference to this question, we can conclude that orthographic functioning in at least one individual is reorganized from the left to right hemisphere, with the possibility of additional perilesional reorganization in this individual.

However, there remain some limitations of the current study. First, the control group in this study is not ideal. CH is compared to a group of young adults. It is possible that the degree of orthographic and visual processing in the right hemisphere may depend on age. In general, aging has been associated with a reduction of hemispheric asymmetry [59]. While this reduction in hemispheric asymmetry has not been shown for reading specifically, it is possible that CH's right hemisphere responds differently to written words than the control group because he is older than the controls, not because he had a stroke. Age-matched control participants could address this concern.

Second, while the similarity index effects reported here are significantly different than zero, they are still exceedingly small. Similarity indices can vary between −1 and 1, but, at best, we observed values around .05 in the current study. These low correlations can partially be explained by noise in the data, but the values obtained in the current study are likely well below the noise ceiling (Nili et al., 2014). Therefore, we conclude that computational models used to compute the similarity matrices are only approximations of the neural computations in the regions that we are investigating. The fact that, in controls, the left VWFA correlates higher with the orthographic than the visual similarity matrix suggests that the orthographic theory is closer to the actual neural computations of that region than the visual theory. However, the bigram model used here to compute orthographic similarity does not fully capture how words are processed in this region, potentially because it is not the correct theory of representation and processing at an orthographic level.

Third, this is a single case study of an individual who, despite having recovered some ability to read words, continues to have visual word processing difficulties well into the chronic state. We would not want to argue from this one case that all patients with acquired dyslexia show contralesional reorganization. For one thing, because this is an exploratory single subject analysis, we did not apply multiple comparisons correction for the ROIs tested. Therefore, it is possible that our results with this one subject reflect a type 1 error. A larger study, with more participants, in which we correct for multiple comparisons, is necessary to draw stronger conclusions about reorganization of the reading system following stroke. Furthermore, it is worth noting that CH has a greater residual reading impairment than many of the other cases whose reading recovery has been studied with fMRI (e.g., [12, 13, 15]). CH's continued impairment many years following the stroke may reflect limits to contralesional reorganization, and patients who make a more complete recovery may benefit from perilesional reorganization rather than contralesional reorganization. There are many open questions about how individual differences in neural plasticity following stroke related to differences in recovery. A large scale case-series investigation using the methods outlined above is necessary to address these questions.

Finally, because the current study is a single case investigation, we choose to select anatomical ROIs based on specific hypotheses about contralesional and perilesional reorganization, rather than functional ROIs based on regions that are more activated in CH than controls in the whole brain univariate analysis. As can be seen in Figure 3, there are a number of anterior regions in which CH shows greater activation than controls: bilateral inferior frontal gyrus and cingulate and insular cortex. One limitation of the current approach is we cannot interpret what this activity means, either in terms of reorganization of function or in terms of engagement of other cognitive processes like cognitive control or working memory. Future research, with a larger population, should apply this same logic of RSA to ROIs selected on the basis of regions in which patients show an increase in activation relative to controls. This approach will be able to interpret what functional changes underlie these increases in anterior activation.

The major contribution of this study is demonstrating how new decoding fMRI techniques can provide stronger evidence for functional reorganization of the reading network following stroke than traditional, univariate activation analyses. These new techniques for analyzing functional neuroimaging data for information, not activation, have proved to be powerful new tools in cognitive neuroscience [60–63]. Studies of language recovery would benefit from using these techniques. These techniques will allow us to map regions that shift their function following damage in a way that univariate, activation-based fMRI simply cannot. This study provides a proof of concept that representational similarity analysis can provide useful insights into functional reorganization following brain damage even with an individual subject. Going forward, we anticipate that representational similarity analysis will play a key role in addressing many of the open questions about the neural plasticity of language recovery: individual differences in neural and behavioral patterns of recovery, the relationship between spontaneous and treatment-induced recovery, and how patterns of recovery differ as a function of the domain of language impairment.

## Figures and Tables

**Table 1:** Range of the visual and orthographic similarity indices values along with CH's value and rank among the 21 participants (20 controls plus CH) for all 7 ROIs reported in the text.

	Visual			Orthographic		
	Control range	CH index	CH rank	Control range	CH index	CH rank
Contralesional VWFA	−.029–.100	−.032	21	−.050–.083	.032	6
Contralesional angular gyrus	−.034–.039	−.037	21	−.041–.053	.057	1
Perilesional fusiform	−.040–.068	−.003	17	−.049–.056	.033	8
Perilesional inferior temporal	−.042–.067	−.009	17	−.051–.085	.022	9
Perilesional medial temporal	−.038–.094	−.025	19	−.031–.067	.026	8
Perilesional anterior temporal	−.040–.057	−.004	11	−.041–.060	.013	11
Perilesional parietal	−.047–.057	.014	9	−.076–.120	.080	3

## Endnotes

1. It is possible that CH's residual word reading is a version of word superiority effect observed in unimpaired readers, whereby briefly presented letters are easier to identify when embedded in words than when embedded in nonwords or shown in isolation [64, 65]. However, CH does not show a word superiority effect on letter identification. His letter naming performance is identical when letters are embedded in words (13/60; 22%) and nonwords (13/60; 22%) and comparable to his isolated letter naming ability (26/104; 25%). Another possibility is that this residual word reading reflects the patient's ability to guess which word is present given degraded, but not fully impaired, letter identity level information (e.g., [21]). This account would predict that CH should be more accurate at reading words with fewer neighbors, as it is easier to guess those words with limited orthographic information. However, when controlling for frequency, imageability, and length, CH's reading ability was not influenced by neighborhood size (many (>5) neighbors = 13/60; 22% versus few (≤1) neighbors = 12/60; 20%), providing evidence against this guessing account.

2. Given the design of our experiment, we cannot distinguish the font-free and case-free levels of representation proposed by Brunsdon et al. [34]. In our experiment, all of the words were presented in the same case, so we were unable to determine whether a given region treated the same abstract letter identity in upper and lower case (e.g., g and G) identically. Given CH's behavioral pattern of impairment, with poor cross-case or cross-modality letter processing but relatively good reading even for words with alternating case, we expect that this orthographic similarity measure will track font-free but not case-free orthographic representations.

3. This procedure is slightly different than that used in other RSA papers. Specifically, other researchers have created representational dissimilarity matrices between two brain patterns by computing 1 − *r*, where *r* is Pearson's correlation between two brain patterns. The choice of Spearman's correlation was motivated by concerns about nonlinearities in the relationship between beta-weights.
